# Variations in Oil Occurrence State and Properties during High-Speed Stirring Treatment of Oily Sludge

**DOI:** 10.3390/toxics12100711

**Published:** 2024-09-29

**Authors:** Yuwei Bao, Yimin Zhu, Yang Liu, Jiao Zhao, Xiaojia Tang, Tie Li, Yin Wang, Xianmeng Liu, Hao Zhang

**Affiliations:** 1 College of Environmental Science and Engineering, Dalian Maritime University, Dalian 116026, China; byw13332259874@dlmu.edu.cn (Y.B.); zhaojiao@dlmu.edu.cn (J.Z.); xiaojiatang@dlmu.edu.cn (X.T.); listeel@dlmu.edu.cn (T.L.); yinwang@dlmu.edu.cn (Y.W.); liuxianmeng@126.com (X.L.); zh1120211935@dlmu.edu.cn (H.Z.); 2 Collaborative Innovation Center for Vessel Pollution Monitoring and Control, Dalian Maritime University, Dalian 116026, China

**Keywords:** oily sludge, oil occurrence state, oil properties, high-speed stirring

## Abstract

Oily sludge (OS) has long been regarded as a hazardous waste, and improper disposal may lead to serious environmental concerns and human health risks. Despite various methods having been proposed and applied to the treatment of OS, the oil occurrence states and properties in sludge are rarely characterized, which may directly link to the selection and effectiveness of treatment methods. Here, confocal laser scanning microscopy (CLSM), X-ray diffraction (XRD), gas chromatography (GC), and four components (SARA) analysis were utilized to characterize the changes in the oil occurrence states and compositions in OS samples before and after high-speed stirring (HSS) treatment. Our results show a substantial reduction in the oil concentration of OS after HSS treatment (from 32.98% to 1.65%), while SARA analysis reveals a similar oil composition before and after treatment, suggesting the broad applicability of HSS in removing oil and its insignificant selectivity towards various hydrocarbon components. This is further supported by the total petroleum hydrocarbon (TPH) analysis results, which show that the separated oil phase has a hydrocarbon composition similar to that of the original OS sample. The CLSM and fluorescence analysis suggest a homogeneous distribution of oil in the sludge, with relatively light components more concentrated in the pore systems between coarse mineral particles, whereas relatively heavy components tend to coexist with clay minerals. After HSS cleaning, both light and heavy components are removed to varying degrees, but light components are preferentially removed while heavy components tend to be retained in the sludge due to adsorption by clay minerals. This is consistent with TPH analysis, where a significant decrease in n-alkanes with lower carbon numbers (n-C14 to n-C20) was observed in the residual sample. Our findings demonstrate the dynamic response of oil occurrence states and compositions to the OS treatment process and highlight the importance of characterizing these fundamental properties prior to the selection of OS treatment methods.

## 1. Introduction

Oily sludge (OS) is inevitably generated throughout most processes of the petroleum industry [1], particularly during petroleum exploitation, transportation, storage, and refining [2]. Based on different sources, OS can be roughly divided into oilfield oily sludge (OOS), tank bottom oily sludge (TBOS) [3], and refinery oily sludge (ROS) [4]. It is estimated that the cumulative amount of oily sludge worldwide exceeds 1 billion tons, and the global petroleum industry accumulates over 60 million tons of oily sludge annually [5]. However, OS has been recognized as a highly hazardous waste due to its toxic and harmful components, including petroleum hydrocarbons (PHCs), polycyclic aromatic hydrocarbons (PAHs), heavy metals, chemical additives, and asphaltenes [6,7]. Therefore, massive discharge and improper disposal of OS may potentially cause serious environmental pollution and threaten human health.

A variety of methods have been established for the treatment of OS, which can be divided into chemical, physical, and biological treatment methods [1,3,8,9]. The commonly used treatment approaches include mechanical separation, froth flotation, centrifugation, ultrasonic and microwave irradiation, solvent extraction, oxidation, pyrolysis, biodegradation, and stabilization/solidification [1,3,4,8]. Among these methods, chemical treatment methods are widely used due to their high adaptability to different types of OS and their high efficiency in removing oil from solids. In traditional chemical treatment procedures, surfactants are commonly used to reduce the interfacial tension between oil and water, thereby promoting the release of oil from solids [7]. However, heating is generally required to disperse surfactants due to the extremely high viscosity of the OS [7]. In addition, a reduction in the interfacial tension of the oil/water interface during oil removal would result in the formation of oil-in-water emulsions, and, hence, other chemicals (e.g., emulsion breakers or reverse emulsion breakers) are required to further treat the emulsions in order to achieve oil–water separation [10,11]. Thermochemical methods such as pyrolysis can provide advantages in energy recovery in addition to reducing sludge volume, but they require a large investment in site and equipment [4]. Biological treatment is considered a green technology that can limit the use of hazardous chemicals and reduce high energy demands and potential secondary pollution [12,13]. However, biological treatment requires a large floor area and a long processing period, which greatly limits the processing capacity [5]. A recent study proposed a high-speed stirring (HSS) treatment method at room temperature, which has the advantages of no heating, high efficiency, and low energy consumption, thus potentially providing a highly economical and effective method for treating OS [14].

Notably, the effectiveness of a given method in treating different types of OS may vary greatly due to the complexity of the sludge in the composition and the oil occurrence state and properties [1,8]. Generally, OS is composed of relatively varying amounts of oil, water, and solid particles [1], which are broadly consistent with the composition of geological petroleum reservoir rocks. In geological petroleum reservoirs such as shale oil reservoirs, crude oil typically exists in the form of free, adsorbed, and dissolved states [15]. Free oil generally appears in macropores and fractures [16], adsorbed oil primarily presents on the surface of organic matter and minerals [17], and dissolved oil mainly exists in kerogen skeleton pores [18]. Under geological conditions, the mobility of these oil types decreases from free oil to dissolved oil due to their differences in composition, density, and viscosity [15]. On the other hand, the distribution of oil with different properties in the reservoir rocks may also have varied to a certain extent, resulting in a strong heterogeneity. For example, studies on reservoir rocks suggest a significant difference in the distribution of light and heavy hydrocarbon components in these rocks, with light components dominating the intergranular pores of brittle minerals (e.g., quartz and feldspar), while heavy components coexist mainly with organic matter and clay minerals [19,20]. In view of the potential similarity between OS and petroleum reservoir rocks at a micro scale, it can be inferred that the differences in the occurrence state and properties of oil in OS may affect the effectiveness of treatment to some extent. However, the heterogeneity in distribution and properties of oil in OS, as well as its response to treatment processes, remains largely unexplored.

Here, our previously developed HSS method was utilized to treat OS samples from the Daqing oilfield in eastern China. Confocal laser scanning microscopy (CLSM) and fluorescence analysis were applied to characterize the occurrence state and distribution of oil in OS. X-ray diffraction (XRD), gas chromatography, and four components (saturates, aromatics, resins, and asphaltenes (SARA)) analysis were used to characterize the chemical composition and properties of OS. More importantly, the combined application of these techniques in pre- and post-treatment OS samples allows us to explore the response of various forms and properties of oil in OS to the HSS treatment process and further reveal the possible mechanisms behind this response.

## 2. Materials and Methods

### 2.1. Samples

The OS samples we examined were collected from Daqing oilfield, China. Prior to the experiment, a 100 mesh sieve was used to remove impurities from the OS. The samples were then evenly mixed and sealed in a cool, low-temperature storage area. All experimental analyses were conducted at the Environmental Science and Engineering Laboratory of Dalian Maritime University, or at the Key Laboratory of Continental Shale Hydrocarbon Accumulation and Efficient Development, Northeast Petroleum University.

### 2.2. High-Speed Stirring (HSS) Experimental Procedure

**Figure 1:**
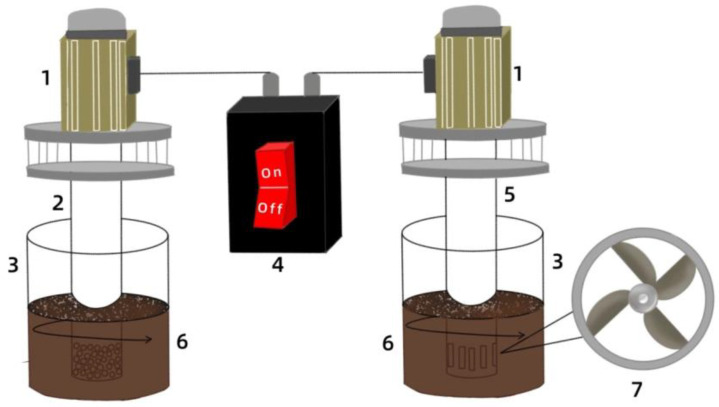
Diagram of a high-speed stirring cleaning device (modified after [14]): 1—brushless motor; 2—Rotor-C; 3—mixing tank; 4—motor controller; 5—Rotor-V; 6—oily sludge; 7—impeller.

The treatment of OS was carried out following the high-speed stirring experimental procedure [14]. First, approximately 200 g of OS and cleaning agent (sodium metasilicate pentahydrate; Tianjin Zhiyuan Chemical Reagent Co., Tianjin, China) is added to the mixing tank at a mass ratio of 25:3, then water is added at a sludge/water ratio of 0.5 g/mL. Subsequently, the motor drives the stirring rotor to rotate at high speeds inside the fixed cylinder, and the OS is circulated and cleaned in the stirring container (Figure 1). Finally, the crude oil floats from the liquid surface and is skimmed, while the residual mixture is centrifuged and dehydrated for oil content measurement.

### 2.3. X-ray Diffraction (XRD) Analyses

The mineral composition of the OS was derived from X-ray diffraction (XRD) analysis. The XRD analysis was performed on a Bruker D8 Advance X-ray diffractometer with Cu-Kα radiation at 40 kV and 30 mA. Stepwise scanning measurements of the samples were carried out at a rate of 4°/min in the range of 3° to 85° (2θ). The relative mineral proportions were estimated from the areas of their major peaks with Lorentz polarization correction [21].

### 2.4. Gas Chromatography (GC) and SARA Analyses

The sample powder was subjected to dichloromethane for 72 h and the soluble organic matter was filtered using extracted cotton. Gas chromatography analysis of the soluble organic matter was carried out on an Agilent 7890 equipped with a HP-5ms fused silica capillary column (30 m × 0.25 mm × 0.25 μm). The oven temperature was initially set at 40 °C and held for 2 min, then raised to 100 °C at a rate of 10 °C/min, then to 300 °C at a rate of 3 °C/min, and maintained at the final temperature for 30 min. Helium was employed as the carrier gas at a flow rate of 1.0 mL/min [22]. For SARA composition analysis, the soluble organic matter was further separated into saturate, aromatic, and resin fraction according to the ASTM D2007 [23]. The insoluble organic matter (asphaltene fraction) was determined by the change in the weight of the filter film before and after use.

### 2.5. Confocal Laser Scanning Microscopy (CLSM) and Fluorescence Analyses

The LEICA SP5II laser confocal microscope was utilized for CLSM analysis in this study. The objective lens is shared by a full-spectrum fluorescence collection and a scanning laser. To produce epifluorescence, a traditional high-pressure mercury lamp was employed with an excitation wavelength of 200–400 nm (ultra violet light). A laser with a consistent excitation wavelength of 488 nm (blue light) was used in this investigation. Two-dimensional (2D) images of the samples were generated by using a laser with a specified wavelength as the launching light source. The three-dimensional (3D) structure of the samples was created through images at various depths within the thin section, which can be obtained by changing the depth of focus [20]. With the increase of thermal maturity and the cracking of hydrocarbons, the fluorescence color generally varies from red, orange, yellow, and green to blue (i.e., blue shift) [24]. Thus, as the maturity and proportion of small molecules (i.e., light components) increases, fluorescence would undergo a ‘blue shift’ and the wavelength peak of the fluorescence spectrum would decrease [25]. Previous CLSM studies on organic matter and oil have shown that the light component typically produces a fluorescence signal in the wavelength range of 490 to 500 nm, while the heavy component is between 680 and 710 nm. Therefore, a fluorescence wavelength of 600 nm is used as a threshold for identifying light and heavy components [10,21].

### 2.6. Oil Content Determination

Approximately 5 g of sludge samples were weighed into a conical flask containing tetrachloroethylene (Tianjin Aoran Fine Chemical Research Institute, Tianjin, China) for extraction and then shaken for 10 min at 200 r/min on a rotary shaker. Subsequently, ultrasound was performed at 40 kHz for 40 min and heated in a water bath at 55 °C for 30 min. After cooling, the sludge was centrifuged at 5000 r/min for 10 min. The oil content in the supernatant was determined by the infrared spectrophotometer.

## 3. Results and Discussion

### 3.1. The Characteristics of OS and the Treatment Effect of the HSS Method

**Figure 2:**
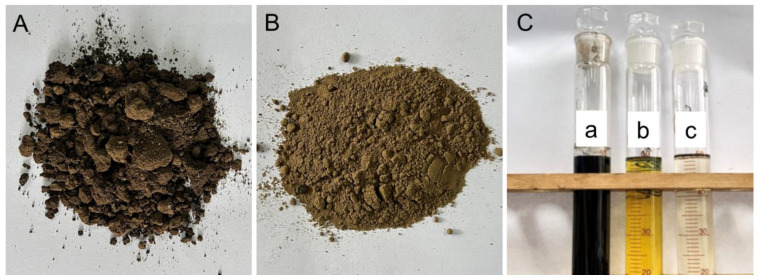
Oily sludge (OS) samples and tetrachloroethylene extraction solution before and after high-speed stirring (HSS) treatment. (**A**) Original OS sample before HSS treatment. (**B**) Residual OS sample after HSS treatment. (**C**) Tetrachloroethylene extract of original OS sample (a), oil phase separated during HSS treatment (b), and residual OS sample (c).

**Figure 3:**
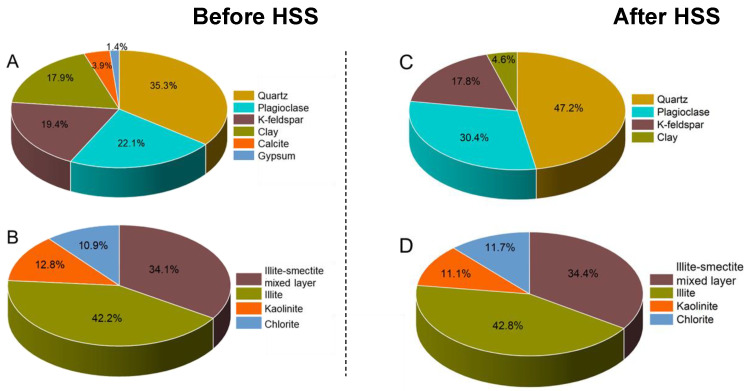
Whole rock mineral (**A**) and clay mineral (**B**) composition of the original OS sample. Whole rock mineral (**C**) and clay mineral (**D**) compositions of HSS-treated samples.

The OS sample used in this study was dark brown to black in color, with no visible crude oil condensate (Figure 2A). The initial oil concentration of the OS sample was 32.98%, and the contents of saturates, aromatics, resins, and asphaltenes in the crude oil were 53.42%, 25.78%, 16.56%, and 4.24%, respectively (Table 1). The identification and quantification of the mineral composition of the OS was obtained through XRD analysis. The mineral composition of the OS sample mainly includes quartz (35.3%), K-feldspar (19.4%), plagioclase (22.1%), and clay minerals (17.9%), with a small amount of calcite (3.9%) and gypsum (1.4%) (Figure 3A). In the clay mineral fraction, illite (42.2%) and illite–smectite mixed layers (I/S) (34.1%) are the dominant clay minerals, followed by kaolinite (12.8%) and chlorite (10.9%) (Figure 3B). The mineral composition of the HSS-treated samples consisted mainly of quartz (47.2%), K-feldspar (17.8%), plagioclase (30.4%), and clay minerals (4.6%) (Figure 3C). In the clay mineral fraction, illite (34.4%) and illite–smectite mixed layers (I/S) (42.8%) are the dominant clay minerals, followed by kaolinite (11.1%) and chlorite (11.7%) (Figure 3D). The XRD data of the samples before and after HSS treatment showed that, in parallel with the removal of oil, the treatment effectively removed clay minerals from the samples to varying degrees, resulting in the observed reduction of clay content and an increase in the relative content of quartz and plagioclase after treatment. This synchronous decrease in oil and clay is likely caused by gravity sorting generated during the HSS processing.

The OS sample was cleaned using our previously established HSS method [14]. After cleaning, the color of the residual sludge changes to light brown (Figure 2B), while the oil concentration decreases to 1.65%. Compared with many other methods, the HSS method has advantages in efficiency, economy, and applicability. For example, thermochemical cleaning is a widely used technique that exploits the lipophilic and hydrophilic nature of hot water and surfactants. This process alters the interaction forces between oil, water, and sludge, significantly reducing the sludge’s viscosity and enabling effective oil–sludge separation [26]. After cleaning, the residual oil rate of the sludge sample is generally between 2–4% [27,28]. With other methods, such as ultrasound-assisted cleaning, the residual oil rate of the cleaned sludge sample can be reduced to less than 2% [29]. However, these traditional methods have drawbacks, including high energy and surfactant consumption, which lead to significant cleaning costs, limited effectiveness, and potential secondary contamination (Table 2). In contrast, the novel high-speed stirring (HSS) method offers a promising alternative due to its economic efficiency, energy savings, and lack of need for additional heating or cooling. Consequently, the HSS method holds potential as a more environmentally friendly and economical sludge treatment technology, poised to bring innovative advancements to the field. A significant reduction in oil content during HSS treatment can also be inferred from the tetrachloroethylene extract (Figure 2C). The extract of the original sample has the darkest color, followed by the extract of the oil phase obtained during the HSS treatment, the lightest is the extract of the residual sample. However, SARA analysis of the residual sample after HSS treatment revealed a crude oil component similar to that of the original sample, with saturates, aromatics, resins, and asphaltene contents of 52.82%, 22.97%, 20.32%, and 3.89%, respectively (Table 1). This suggests that the HSS treatment has a broad applicability for oil phase separation in OS, and the treatment effect of OS may have been mainly controlled by the oil occurrence state. We then explored this possibility by conducting in situ observation of the oil phase in OS using CLSM and fluorescence analyses.

### 3.2. Variations in Oil Occurrence State and Properties during Treatment

**Figure 4:**
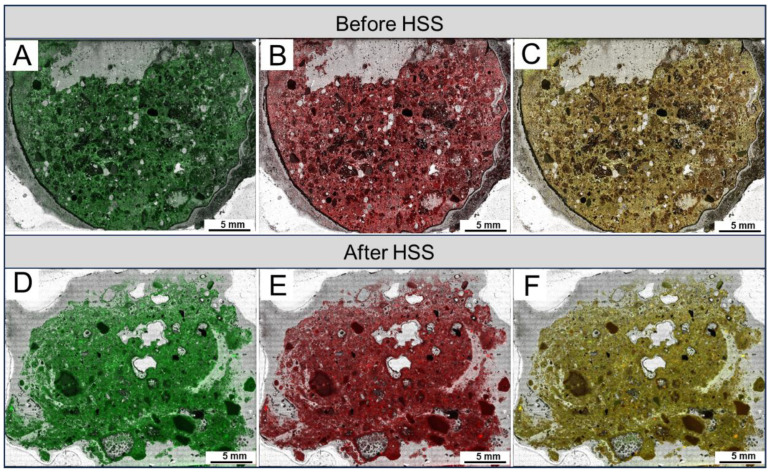
Large-field imaging of OS samples by confocal laser scanning microscopy (CLSM) before (**A**–**C**) and after (**D**–**F**) HSS treatment. (**A**,**D**) Light component. (**B**,**E**) Heavy component. (**C**,**F**) Overlapping images of light and heavy components.

The integrated CLSM and fluorescence detection techniques were used in this study to characterize the changes in the occurrence states of oil in sludge during HSS treatment. Compared with conventional optical microscopy and scanning electron microscopy (SEM), the application of the CLSM method in OS characterization has the following advantages. First, the distribution of different oil phases (light and heavy components) in OS samples can be observed. Second, more detailed information about pore structures and oil occurrence states in OS samples can be obtained through multi-layer scanning and 3D reconstruction [19,20,34]. The confocal large field imaging shows that the distribution of light (colored green) and heavy (colored red) components in OS is relatively homogeneous with no significant differential enrichment, both before and after HSS treatment (Figure 4). This can be seen from the overlapping images of light and heavy components, where a relatively uniform yellow-green color predominates and is accompanied by local variations. Lighter green represents more enrichment of light components, and darker orange indicates more enrichment of heavy components. Although the distribution of oil does not seem to be significantly affected by HSS treatment, the overall signal of oil in the treated sample is weakened (lighter in color) compared to the pre-treated sample (Figure 4), suggesting a high efficiency of the HSS treatment in oil separation from the solid phase.

**Figure 5:**
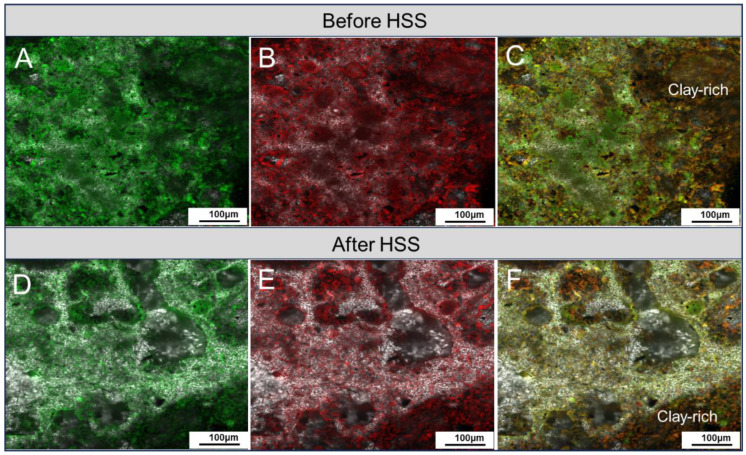
CLSM imaging of OS samples obtained using a 20× objective lens with microscope before (**A**–**C**) and after (**D**–**F**) HSS treatment. (**A**,**D**) Light component. (**B**,**E**) Heavy component. (**C**,**F**) Overlapping images of light and heavy components.

**Figure 6:**
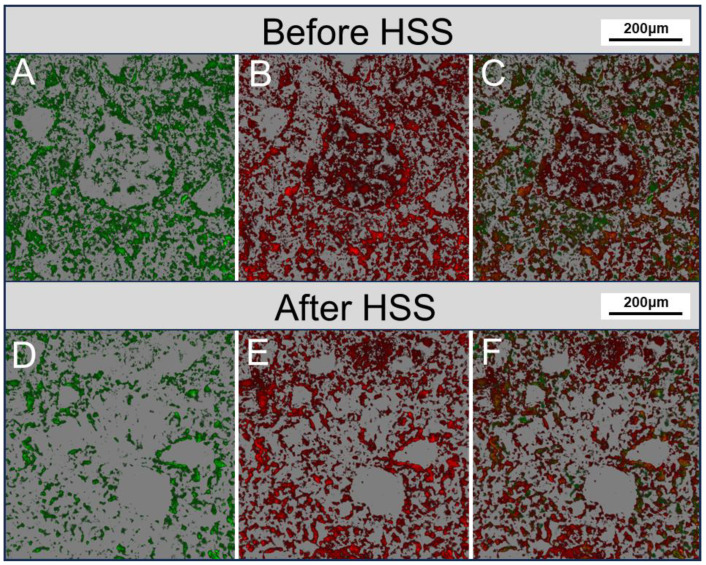
Microphotographs showing the 3D reconstruction of samples based on CLSM before (**A**–**C**) and after (**D**–**F**) HSS treatment. (**A**,**D**) Light component. (**B**,**E**) Heavy component. (**C**,**F**) Overlapping images of light and heavy components.

At a larger magnification, the inter-pores between coarse mineral particles typically appear green in fluorescence, indicating a greater enrichment of light components. In contrast, heavy components coexist primarily with clay minerals or are concentrated at the edges of mineral particles (Figure 5). The comparison of samples before and after HSS treatment shows that both light and heavy components were removed to varying degrees during the HSS treatment process. However, the decrease in heavy components seems to be smaller, resulting in more residues in sludge, particularly those coexisting with clay minerals. This is further supported by the 3D reconstruction of OS samples, which provides more detailed information on the occurrence states of oil in the pore systems of the sludge matrix. The 3D reconstruction of the samples before and after HSS treatment shows a clear variation in oil concentration, with light components largely removed and more heavy components retained in the sludge (Figure 6). Two possible mechanisms can be invoked to explain the observed results. First, oil that occurs in pores between coarse mineral grains may have higher mobility, either due to larger pore diameters or to lighter oil properties. Second, the strong adsorption capacity of clay minerals for oil may have hindered the separation of oil from the solid phase. Indeed, clay minerals are thought to have a high affinity for oil, which would facilitate oil adsorption and lead to a decrease in their fluidity [35]. In sedimentary environments, clay minerals have been widely recognized to play an important role in promoting the preservation of organic matter [36,37]. In addition, clay minerals are thought to have selective adsorbability for high molecular weight, aromatic, and aliphatic compounds [38,39]. Once retained by clay minerals, the stability of these compounds would be enhanced and therefore resistant to degradation or desorption [39,40].

**Figure 7:**
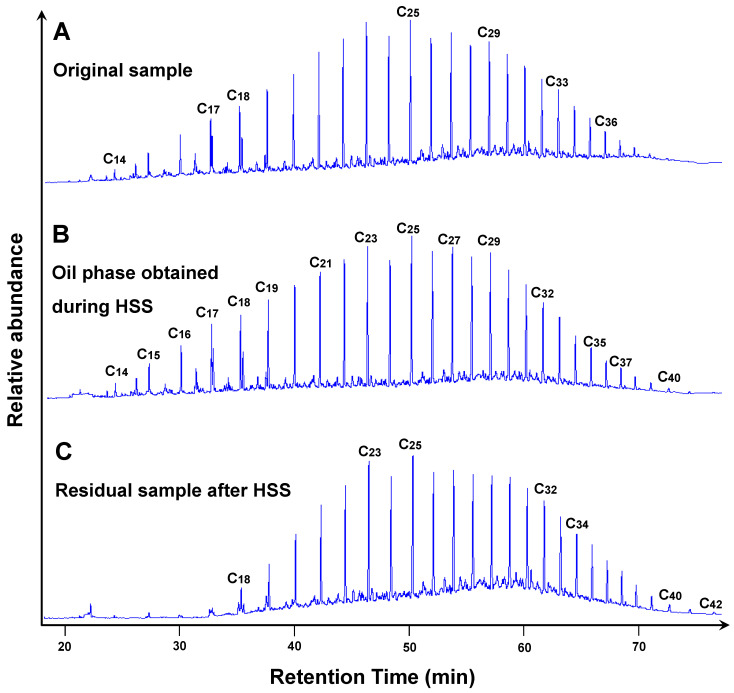
Chromatograms of total petroleum hydrocarbon (TPH) in the original OS sample (**A**), oil phase separated during HSS treatment (**B**), and residual sample (**C**).

We then explored the variations in oil composition during HSS treatment using gas chromatography. The original OS sample, oil phase separated during HSS treatment, and residual sample were collected for total petroleum hydrocarbon (TPH) analysis (Figure 7). The GC chromatogram of the original OS sample shows that n-alkanes are the dominant compounds in the oil. The n-alkanes range from n-C14 to n-C40, and are dominated by high-molecular components with an evident odd carbon number preference in the C23–C39 region. The GC chromatogram of the separated oil phase during HSS treatment shows a pattern similar to the original sample, suggesting that HSS cleaning is generally effective for a broad range of oil components in OS without significant compound selectivity. The GC chromatogram of the residual sample demonstrates that the residual oil is still predominantly composed of n-alkanes, consistent with the results from SARA analysis. However, compared to the original sample, the residual sample exhibits a significant loss of compounds with lower carbon numbers (n-C14 to n-C20), suggesting that relatively lighter components were preferentially removed during HSS treatment, consistent with our inference from CLSM observations. In addition, a significant shift in unidentified complex mixture can be observed in the GC chromatogram of the residual sample, suggesting that the presence of unidentified compounds became evident as the dominant n-alkanes were largely removed during HSS treatment.

### 3.3. Implications for the Treatment of OS

The environmental hazards and resource recovery potential of OS have received widespread attention in the past few decades [41]. The degradation of organic materials and compounds found in oily sludge is challenging, thereby complicating the assessment of treatment efficiency [42], and various treatment methods and technologies have emerged as a result [7,43]. However, the occurrence state and nature of oil can vary considerably, largely depending on the type of OS. For example, oilfield oily sludge (OOS) is produced during oil exploitation and drilling and is therefore a mixture of oil, drilling mud, soil, and water [9,44]. In contrast, tank bottom oily sludge (TBOS) is formed during the crude oil storage process [45], which is characterized by high oil content [46], a large amount of soluble inorganic salts, and a predominance of heavy oil components [4]. Therefore, differences in the composition and fundamental nature of different types of OS may affect the effectiveness of a particular OS treatment approach. For example, the application of a physical treatment method (such as ultrasound) on TBOS may be expected to be less efficient due to the high proportion of heavy oil components and very fine-grained clay-rich sediments in TBOS [47,48]. Indeed, despite the fact that the HSS method used in this study was combined with the addition of surfactants, our data show that the HSS method appears to be closely related to the clay mineral content in the sludge and the composition of petroleum hydrocarbons, which controls the occurrence state and distribution of oil in the sludge. The oil present in larger scale pore systems is preferentially removed during HSS treatment, while the relatively heavy components bound to the clay minerals are not effectively removed due to the strong adsorption of clay to oil. The distribution and migration behavior of oil in sludge are similar to those in geological petroleum (particularly shale oil) reservoirs [20,49].

It has been proposed that many oily pollutants may not be effectively treated through a single treatment method, and, hence, a combination of several methods is generally required [1,9]. On the other hand, the advantages and disadvantages of various treatment methods are generally evaluated from aspects such as treatment efficiency, energy consumption, investment, and operating costs [9], without fully considering the applicability of methods from the perspective of the inherent properties of OS. Our study suggests a dynamic response of oil occurrence states and properties to a specific OS treatment method, highlighting the need for comprehensive consideration of intrinsic properties of OS beyond the aforementioned factors. Hence, our study potentially provides new insights into the selection strategy of OS treatment methods.

## 4. Conclusions

This study reports a combination of XRD, GC, and SARA data and CLSM observations for OS samples before and after HSS treatment. Results demonstrate a considerable drop in OS oil concentration (from 32.98% to 1.65%) following HSS treatment, and SARA analysis shows a comparable oil composition before and after treatment, indicating the broad applications of HSS in oil removal and its negligible selectivity towards different hydrocarbon components. This is consistent with TPH results from GC analysis, which show that the hydrocarbon composition of the separated oil phase is identical to that of the original OS sample. The CLSM and fluorescence observations point to a homogenous distribution of oil in the sludge, with relatively light components more concentrated in the pore networks between coarse mineral particles and comparatively heavy components typically coexisting with clay minerals, either adsorbed onto the mineral surface or present in the micropores of clay minerals. Both light and heavy components are removed to varying degrees during HSS cleaning; however, heavier components tend to be retained in the sludge because of their adsorption by clay minerals, whilst light components are more intensively removed. This is in line with the results of the TPH analysis, which shows that the residual sample had a considerable decline in n-alkanes with lower carbon numbers (n-C14 to n-C20). These findings provide evidence for the dynamic response of oil occurrence states and compositions to the OS treatment process and suggest that these fundamental characteristics may potentially affect the effectiveness of specific OS treatment methods, which need to be taken into account when selecting OS treatment methods.

## Figures and Tables

**Table 1:** Oil concentration and SARA composition of OS samples before and after HSS treatment.

Samples	Oil Concentration (%)	SARA Composition			
		Saturates (%)	Aromatics (%)	Resins (%)	Asphaltenes (%)
Before HSS treatment	32.98	53.42	25.78	16.56	4.24
After HSS treatment	1.65	52.82	22.97	20.32	3.89

**Table 2:** Comparison of oily sludge treatment methods.

Treatment	Advantages	Drawbacks	Oil Removal Rate
Centrifugation treatment	Wider adaptability, good reduction, and combustion to produce heat.	Waste of petroleum resources, easy to cause secondary pollution.	92–96% [30].
Solvent Extraction	The crude oil recovery rate is high, and the extractant can be recycled.	Higher cost, larger amount of extractant.	76% [31].
Pyrolysis	Simple operation, high processing efficiency, and high crude oil recovery rate.	High energy consumption, more complex equipment.	70–84% [32].
Freeze/thaw treatment	High environmental protection; no secondary pollution.	Strict reaction conditions; long processing time; high technical requirements.	50% [33].
High-speed stirring (HSS) method	More environmentally friendly and economical, bringing innovative advances to the field.	Subsequent recycling of oil sludge has not been explored.	95% [14].

## Data Availability

All data are included in the manuscript.
